# The association between *E*. *coli* exceedances in drinking water supplies and healthcare utilisation of older people

**DOI:** 10.1371/journal.pone.0273870

**Published:** 2022-09-01

**Authors:** Gretta Mohan, Seán Lyons

**Affiliations:** 1 Economic and Social Research Institute, Dublin, Ireland; 2 Department of Economics, Trinity College, Dublin, Ireland; Purdue University, UNITED STATES

## Abstract

Evidence concerning the effects of indicators of waterborne pathogens on healthcare systems is of importance for policymaking, future infrastructure considerations and healthcare planning. This paper examines the association between the detection of *E*. *coli* in water tests associated with drinking water supplies and the use of healthcare services by older people in Ireland. Uniquely, three sources of data are linked to conduct the analysis. Administrative records of *E*. *coli* exceedances recorded from routine water quality tests carried out by Ireland’s Environmental Protection Agency are first linked to maps of water systems infrastructure in Ireland. Then, residential addresses of participants of The Irish Longitudinal Study of Ageing (TILDA), a nationally representative survey of over 50-year-olds in Ireland, are linked to the water systems dataset which has the associated water quality monitoring information. Multivariate regression analysis estimates a greater incident rate ratio (IRR) of General Practitioner (GP) visits in the previous year where *E*. *coli* is detected in the water supply associated with an older person’s residence (Incidence Rate Ratio (IRR) 1.118; [95% Confidence interval (CI): 1.019–1.227]), controlling for demographic and socio-economic factors, health insurance coverage, health, and health behaviours. Where *E*. *coli* is detected in water, a higher IRR is also estimated for visits to an Emergency Department (IRR: 1.292; [95% CI: 0.995–1.679]) and nights spent in hospital (IRR: 1.351 [95% CI: 1.004–1.818]).

## 1. Introduction

Exposure to water borne pathogens in drinking water can result in serious illness and a need for the use of healthcare services [1–4]. Gastrointestinal illness is a particular burden for the elderly: studies have demonstrated that hospitalisation and case-fatality rates for gastrointestinal illness among older people are disproportionately high [5–7]. Gastrointestinal illness, including diarrhoeal disease, have been linked to *Escherichia coli* (*E*. *coli*) detected in drinking water [8].

The aim of this study is to examine the association between the detection of *E*. *coli* in drinking water supplies and the use of healthcare services by older people in the study setting of Ireland. The motivation for this research stems from an absence of evidence on the association between pollutant limit exceedances in drinking water tests and the use of healthcare services in extant international literature. An understanding of such links may assist in policymaking, environmental and healthcare management, and infrastructural planning in these areas. Moreover, the combination of greater risks of waterborne diseases presented by climate change, combined with ageing of the population in developed countries, could mean that morbidity and mortality linked to waterborne diseases may be expected to rise in many jurisdictions. We undertake a novel approach to understanding these processes where we spatially link individual-level survey data on health outcomes and socioeconomic characteristics of older people resident in Ireland to geocoded Environmental Protection Agency (EPA) data on quality of the drinking water supplied to respondents’ residences.

Levallois and Villanueva [9] remark that despite improvements in recent decades, access to good quality drinking water remains a critical public health issue. Drinking water guidelines are issued by the World Health Organization [10], where access to safe drinking-water is regarded as essential for human health, a human right, and a component of health protection policy. The elderly are identified as a vulnerable subpopulation at substantial risk of disease, severe illness, and mortality from exposure to waterborne pathogens. The European Union (EU)’s Drinking Water Directive, provides the legislation for ensuring that drinking water is safe and regulated effectively in Member States [11]. Under this legislation, water quality monitoring must test for *E*. *coli* as a microbiological parameter providing an indication of faecal pollution [12], and the detection of *E*. *coli* in water is recognised as an indicator of waterborne disease.

Pathogenic microorganisms such as *E*. *coli* bacteria may be ingested while drinking contaminated water or by eating food prepared with contaminated water. Neumann [13] notes while that the presence of microorganisms in water cannot be used as predictors for disease outbreak, they provide an indication of water quality. Moreover, the authors point out that while the frequency of water quality failure may be low for regulated community water systems, the impact of a single failure can be dramatic, as demonstrated by large scale water borne disease outbreaks originating from regulated water systems in Europe and North America [14–18].

Greater interest in the role of *E*. *coli* as cause of gastrointestinal illness in developed countries has developed with the emergence of E. coli O157:H7, and other *enterohaemorrhagic E*. *coli* (EHEC) such as O26, as significant risks to public health. The increasing risk from water-borne pathogens in developed countries has been attributed to a range of factors including population growth, greater urbanisation, the interaction of farming practices with rainfall, climate change, increases in the number of immunocompromised individuals, drug resistance and genetic changes in strains of microorganisms such as *E*. *coli* [13, 19].

Symptoms of EHEC infection include abdominal cramps and pain, diarrhoea, in some cases vomiting, and this can progress to bloody diarrhoea. The development of haemolytic uremic syndrome (HUS) from EHEC infection can be life threatening, and is particularly serious for young children and older people [20–22]. Reilly [20] reports that on average, 2–7% of patients with HUS die, but for some outbreaks among the elderly the mortality rate has been as high as 50%.

The pathogen *E*. *coli* O157:H7, is associated with *Verocytotoxigenic Escherichia coli* (VTEC) infection, and has been found to be associated with hospital episodes of older people [23, 24]. In Ireland, the country on which this study focuses, one-in-ten cases of the O157 strain occurred among those aged 65 and over [25]. Ireland has among the highest cases of water related disease in Europe due to intense farm activity and the widespread use of private wells [25–32]. Cattle, sheep, and pigs are vectors of *E*. *coli* O157 where the bacteria are shed in the faeces. Waterborne transmission, associated with exposure to contaminated water (from untreated or poorly treated private water sources), is the second most common reason for VTEC infection, and exposure is particularly pronounced following periods of heavy rainfall. More broadly, the quality of drinking water is recognised as an important environmental determinant of health in domestic Irish policy such as *Healthy Ireland* [33]. A number of enteric diseases that may be transmitted by the waterborne route including VTEC infection are legally notifiable under the Infectious Disease Regulations [34], requiring a Medical Officer of Health to identify and remove the causes of disease.

Extreme weather events resulting from climate change are a significant trigger for contamination of public and private water supplies, increasing the risk of human waterborne infections [29]. Forzieri et al. [35] projects that Ireland will be the second most affected European country in terms of the proportion of the national population likely to reside in flood prone areas in 2100, and is thus susceptible to flood associated health impacts [36]. P Philipsborn et al. [37] estimate an 8% increase in the incidence of diarrheagenic *E*. *coli* for each 1°C increase in mean monthly temperature, underscoring the need to intensify efforts to prevent the transmission of these pathogens, and the consequences of these for vulnerable groups such as older people.

On balance, there is a relatively sparse evidence base which considers the association between water borne illness among older people, and still less that examines effects on healthcare utilisation for this group. This study aims to contribute the international literature in this increasingly important area using a novel data linkage approach, described in the next section. We hypothesize that the detection of *E*. *coli* in the water will have a positive association with healthcare utilisation, where we expect an increase in GP visits, ED visits and hospital nights for older people where *E*. *coli* is present in drinking water supplying their homes.

## 2. Methods

In this study we combine individual-level data from the year 2010 on healthcare utilisation and socioeconomic characteristics of a large sample of older people linked to drinking water quality test results taken from the water schemes connected to respondents’ residences. Linkage is made possible by the presence of spatial identifiers both for study participants and for the locations of monitored water schemes.

### 2.1. Data

To examine the extent to which *E*. *coli* in water may be associated with older people’s utilisation of healthcare in Ireland three sources of data are linked for this investigation. These sources are described in turn. We note that we use data collected for the year 2010 from the datasets for linkage (that of the EPA’s water quality monitoring data and the TILDA data) as they most accurately align with the 2009 water infrastructure maps available for this research. More recent water infrastructure maps would allow for linkage of more recently collected data, which is an avenue for future research.

#### 1. Drinking water monitoring data

The EPA keeps records of drinking water monitoring results and water supply details for Ireland (in the SAFER archive), which contain information on microbiological and chemical standards for water supplies. Microbiological information includes test results for bacteria (e.g., *E*. *coli*, *Enterococci*), and chemical information includes metal content (e.g., lead), pesticides and other pollutants. Test results showing unacceptable levels of bacteria or chemicals are known as “pollutant exceedances” (of regulatory limits set for European standards), and information about these exceedances is available for the year 2010. The EU drinking water quality limit for *Escherichia coli* is a zero count of *E*. *coli* detectable per 100 millilitres of water. Records are available by water scheme and date, and the EPA produces annual reports of water quality on monitored supplies (see [38–40]). Based on the EPA data, we create a proxy variable for water quality problems that take the value of 1 if an area’s water source has had *E*. *coli* detected in the water in the year 2010 and 0 otherwise. Schwarzenbach et al. [41] comments that it is “…becoming current practice to rely exclusively on the presence/absence of *E*. *coli* to judge the hygienic quality of drinking water…”, where “…the detection of *E*. *coli* will remain the hygienic parameter for the next decades”.

**Fig 1:**
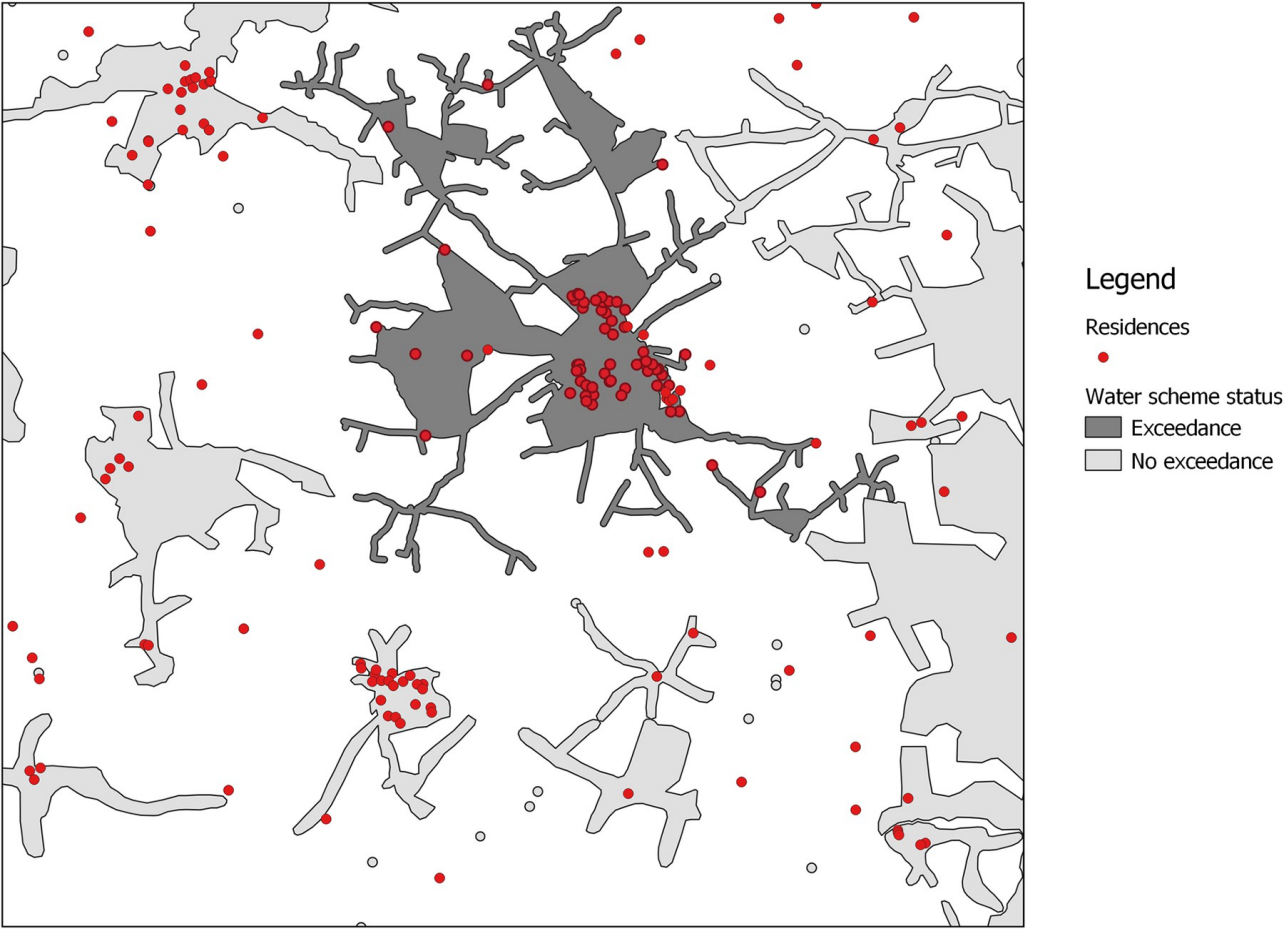
Hypothetical worked example of mapping of *E*. *coli* exceedance, water supply and residence.

Small private drinking water sources such as wells are exempt from the relevant regulations. These private groundwater supplies serve less than 50 persons and/or extract less than a volume of 10 cubic metres per day and are thus exempt from the European Commission Drinking Water Directive, where owners are not subject to legal obligations to test or treat supplies. There is no EPA record of monitoring of water quality on these private sources and thus there is no data for *E*. *coli* detection for such sources. However, we can include respondents connected to such sources in the model by using a 1/0 indicator variable for them. This does not distinguish between respondents whose water contained pollutants and those that did not, but it does pick up the average association between being on a non-tested water source and our outcome variables. S1 Fig 1 in the S1 File shows where *E*. *coli* exceedances have been recorded across the Republic of Ireland, as well as areas for which tests are not recorded.

The EPA water quality report for 2010 [38] indicates that the majority of drinking water in Ireland came from public water supplies (84.8%), serving over 3 million people, with the remainder provided by group water schemes and private supplies (including wells serving single houses). For 2010, 59 boil water notices were active, relating to 51 supplies; 43 of which were new (serving over 66,000 persons). The EPA were notified (by local authorities) of the detection of *E*. *coli* in public water supplied on 46 occasions in 2010.

#### 2. Microdata on healthcare utilisation and socioeconomic characteristics

Data from the first wave of The Irish Longitudinal Study of Ageing (TILDA) collected in the period 2009–2011 are used for this study. TILDA is a nationally representative survey of those aged 50 years or older living in the Republic of Ireland. The study captured demographic, socio-economic, family and health information on 8,175 participants 50 years and above living in residential households at baseline, achieving an 62% response rate. A computer-assisted personal interview (CAPI) was conducted with participants face-to-face in their homes. Participants were also given a self-complete questionnaire (SCQ) which contained an array of additional, more sensitive questions concerning their wellbeing and experiences.

#### 3. Map of water systems

Prior to the Water Services Act in 2013, water and wastewater services in Ireland were provided by water service authorities in 31 local authorities. The map of water supply infrastructure for this study was for 2009 from the Local Government Computer Services Board, a shared-services provider. Under the Act, these services were later brought together under one national utility, Irish Water. The residential addresses of TILDA respondents were linked to water schemes from this map. Where a residential address was not linked to this map, these addresses were marked as ‘unmapped’, and we may assume their drinking water supply is from a private source e.g., a well. Using Geographic Information System software (QGIS v3.10), exceedances of *E*. *coli* from the 2010 EPA water quality data were associated with the 2009 water infrastructure map.

Using QGIS, the residential address of each TILDA respondent was mapped to the relevant water scheme and the scheme was linked (via a scheme code) to information on the presence or absence of *E*. *coli* exceedances. This matching exercise yielded a 1/0 indicator variable marking *E*. *coli* exceedances among respondents on water schemes subject to testing and a second 1/0 indicator variable showing respondents not mapped as being on monitored schemes. Fig 1 is a hypothetical worked example representing the spatial linkage process using constructed data. The illustration distinguishes between residences on monitored schemes with no *E*. *coli* exceedance (light grey polygons), those with a measured exceedance (the dark grey polygon) and those not on a monitored scheme (not within a polygon).

### 2.2. Modelling strategy

This paper investigates the association of *E*. *coli* detection in water supply and three healthcare utilisation outcomes of interest–GP visits, ED visits and hospital nights. In the Andersen framework of determinants of healthcare utilisation [42, 43], the detection of *E*. *coli* in drinking water supply pertains to the ‘need’ factor for healthcare use, where illness caused by ingestion of a pathogen, such as gastrointestinal difficulties among older people, precipitates a need for services.

#### 2.2.1. Outcome

Three outcomes are available for study from TILDA for this investigation: the reported number of visits to a GP in the previous 12 months, the number of visits to hospital ED in the previous 12 months and number of nights in hospital in the previous 12 months. Where respondents have reported an extreme use of services, cases exceeding three standard deviations from the mean of the three outcomes are identified and dropped from the analysis.

We also create a dummy to indicate whether the respondent had visited the GP in the previous year = 1 where one visit or more is reported, = 0 if no visits are reported, and similarly, a dummy for whether a respondent has visited an ED in the previous year, and a dummy for whether they had spent at least one night in hospital.

#### 2.2.2. Exposure of interest

The binary *E*. *coli* exceedance variable is the exposure of interest in this study, = 1 where *E*. *coli* was detected in the water in 2010 in the water supply likely to supply a TILDA respondent’s home, = 0 where *E*. *coli* was not detected in the water supply, or for which there was no water monitoring data for the scheme or for which the residential address was not mapped. Dummies were included models included to indicate whether the TILDA residence was not mapped.

We hypothesize that the detection of *E*. *coli* in the water will have a positive association with healthcare utilisation, where we expect an increase in GP visits, ED visits and hospital nights for older people where *E*. *coli* is present in drinking water supplying their homes.

#### 2.2.3. Modelling approach

The nature of the outcome variables measure is discrete and non-negative, and the variance of each outcome is greater than the mean (see Table 1), requiring a negative binomial regression model count data approach. The estimated negative binomial model of the outcomes may be expressed as:

$$Pr(y_{i}=y_{i}^{*})=\frac{\Gamma (y_{i}^{*}+\upsilon )}{\Gamma (y_{i}^{*}+1)\Gamma (\upsilon )}{\left(\frac{\upsilon }{v+u_{i}}\right)}^{\upsilon }\times {\left(\frac{u_{i}}{v+u_{i}}\right)}^{y_{i}^{*}},y_{i}^{*}=0,1,2,\ldots$$ (1)

where Γ is the gamma distribution function, *y_i_* is the number of GP/ED visits or hospital nights, with $u_{i}=exp(x_{i}\beta ),\upsilon =\alpha^{-1}exp(x_{i}\beta ),\;x_{i}$ is the vector of explanatory variables described below, and *β* is the estimated effect of the explanatory variable of interest.

**Table 1:** Characteristics of sample.

*Observations*	7,643					
**Outcome**						
	**Mean**		**Standard deviation**	**Variance**	**Minimum**	**Maximum**
*GP visits*	3.6		3.5	12.2	0	20
*ED visits*	0.17		0.47	0.22	0	3
*Hospital nights*	0.15		0.44	0.19	0	3
			**Percent (%)**			
*Had GP visit*			87.3			
*Had ED visit*			13.7			
*Had hospital night*			11.7			
***Exposure of interest*: *E*. *coli exceedances***						
*E*. *coli exceedances*			4.0			
*Not mapped*			21.5			
***Covariates***						
*Gender*		Male	45.8			
		Female	54.2			
*Age*		Mean	63.7			
*Age category*		50–59	40.4			
		60–69	32.0			
		70+	27.6			
*Marital status*		Married	69.7			
		Not married: never married, separated, divorced, widowed	30.3			
*Highest educational attainment*		Primary	30.0			
		Secondary	40.3			
		Tertiary	29.7			
*Employment status*		Retired	37.0			
		Employed/self-employed	36.7			
		Unemployed	4.9			
		Looking after home/family	15.6			
		Other	5.8			
*Medical card*		Medical card	46.8			
		No medical card	53.2			
*Private health insurance*		Private health insurance	41.8			
		No private health insurance	58.2			
*Self-rated health*		Good/very good/excellent	78.2			
		Fair/poor	21.8			
*Any Instrumental Activities of Daily Living (IADL) impairment*		Has IADL impairment	11.0			
		No IADL impairment	89.0			
*Depression status*		Depression symptoms	9.2			
		No depression	90.8			
*Smoker*		Smokes	18.0			
		Non smoker	82.0			
*Physical activity level*		High	34.2			
		Medium	34.8			
		Low	31.0			
*Number of regular medicines*		0	28.8			
		1–2	30.3			
		3–4	20.9			
		> = 5	19.9			

Separate regressions models are estimated for each outcome. The most basic model, Model 1, is a univariate model relating an outcome to *E*. *coli* exposure (*x_i_*) alone. A second model, Model 2, adjusts Model 1 to include additional explanatory variables including gender, age, age squared, married, education attainment, employment status (as retired, employed/self-employed, unemployed, looking after home/family, other), entitlement to free public healthcare (referred to in Ireland as a ‘medical card’) and private health insurance status. Model 3 further adjusts model 2 for health status, including reported self-rated health (as good or better), any difficulties with instrumental activities of daily living (IADL) and depression status (as measured by Center for Epidemiologic Studies—Depression Scale (CESD-8)). Model 4 further adjusts Model 3 to account for several health behaviours including smoking status, level of physical activity, and a categorisation of the number of regular medicines taken by the respondent.

To maintain a comparable sample for the analyses across the outcomes, TILDA respondents with missing values of the included variables are dropped. The final analytical sample size comprises of 7,643 TILDA participants. Standard errors are clustered at the TILDA household level to account for study participants who live in the same household. The null may be rejected where empirical estimates meet the statistical significance threshold of a p-value of p<0.05. All analyses are conducted in STATA v.15.

## 3. Results

Summary statistics are presented in Table 1. The mean number of visits to a GP in 12 months was 3.6 visits, with 87.3% of the sample having at least one visit to the GP in the previous year. The average number of visits to ED and hospital nights was 0.2; fewer than one in six respondents attended ED or had a hospital night (13.7% and 11.7% respectively). Four percent of the sample was on a water supply with an *E*. *coli* exceedance, while 96% of the sample of TILDA respondents did not have an *E*. *coli* exceedance detected for 2010. Over a fifth of the sample (21.5%) was not located on a monitored water supply.

In terms of covariates, there are more women than men in the sample, and the majority have secondary education as their highest educational attainment. There was variation in the sample across different states of employment, slightly less than half (46.8%) had entitlement to free public healthcare (a ‘medical card’), and two in five had private health insurance (41.8%). Most of the sample rated their health as good or better (78.2%), though one in ten reported problems with instrumental activities of daily living (11.0%) and 9.2% met the threshold for depression according to the CESD-8 measure. Most of the sample were non-smokers (82.0%), the sample was evenly split across the physical activity categories, and the largest proportion of the sample reported taking 1–2 regular medicines (30.3%).

**Fig 2:**
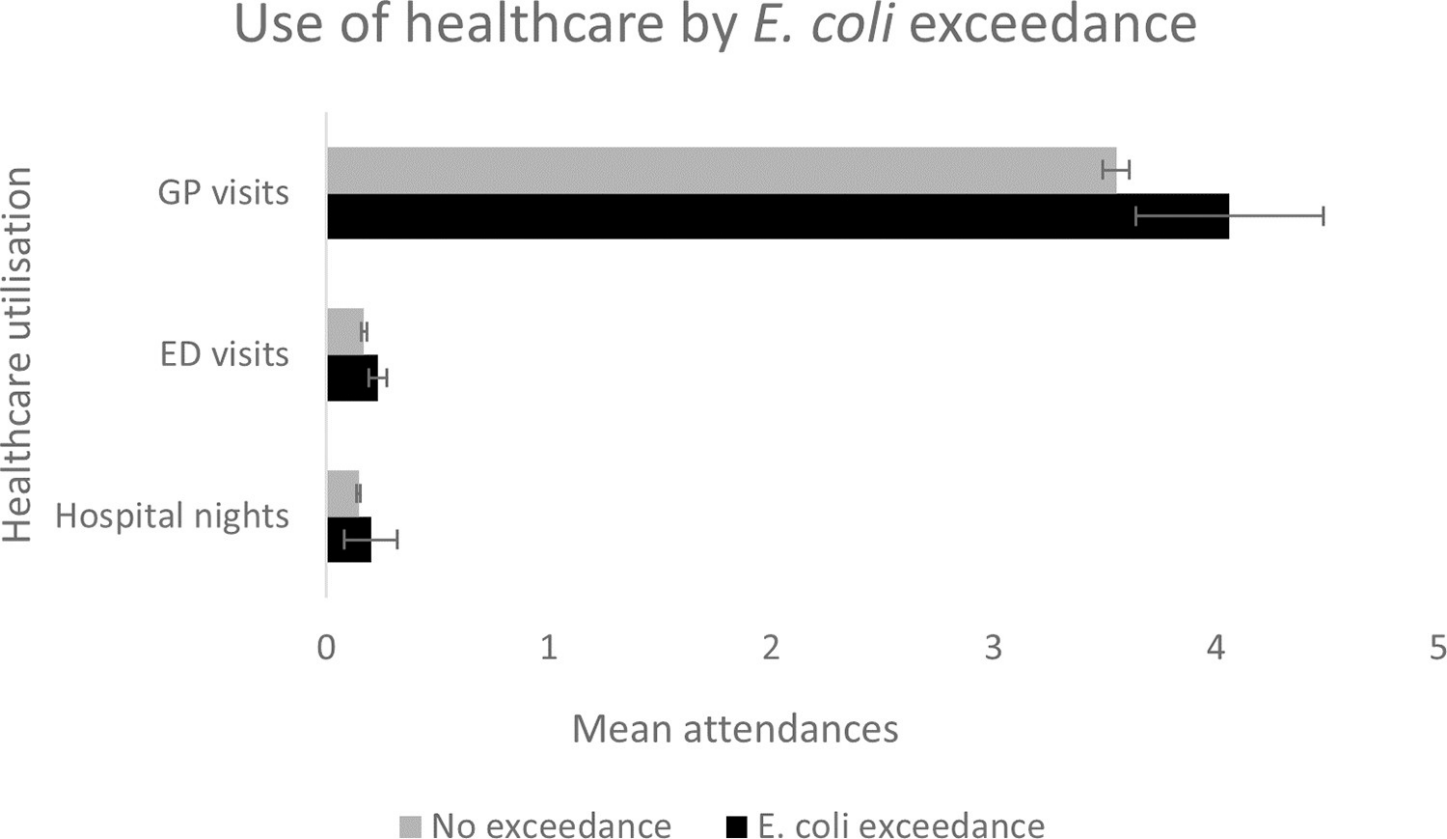
Use of healthcare by *E*. *coli* exceedance during preceding year at respondent’s residence.

Fig 2 shows that the mean number of GP visits for those for whom *E*. *coli* was detected in the water supply was slightly higher than for those for whom this was not an issue, and this pattern is replicated for mean number of ED visits and hospital nights. Two-sample t-tests of the outcomes by whether an *E*. *coli* exceedance was detected or not, estimate a significant difference in GP visits (mean difference -0.51, t = -2.49, p = 0.013), ED visits (mean difference -0.06, t = -2.30, p = 0.021), and hospital nights (mean difference -0.06, t = -2.17, p = 0.030). To allow for potentially confounding socioeconomic, health and behavioural factors, we turn next to regression analysis.

Regression results testing the associations between use of healthcare services and *E*. *coli* exceedance in the drinking water supplies are presented in Table 2 (full results are shown in S1 Tables 1–3 in the S1 File). Across all model specifications, a statistically significant effect of exposure to *E*. *coli* on the number of GP visits over the previous 12 months was estimated. In the most adjusted specification, Model 4, the incidence rate ratio (IRR) of 1.118 [95% Confidence interval (CI): 1.019–1.227], indicates that for those for whom *E*. *coli* was present in their water supply, they had 1.1 times the rate of GP visitation compared to those for whom there was no evidence of *E*. *coli* in their water. Compared to those for whom there was no evidence of issues with the water supply, an *E*. *coli* exceedance was associated with 1.3 times the rate of an ED visit (IRR: 1.292 [95% CI: 0.995–1.679]) in the most adjusted model, though the p-value of p = 0.055, is greater than that of the statistical significance threshold of p<0.05. The detection of *E*. *coli* in water supplies was associated with a 1.3 times rate of hospital nights (IRR: 1.351 [95% CI: 1.004–1.818]). Where a respondent was not mapped onto a water system (and thus probably using a well), this did not have a statistically significant association with healthcare utilisation.

**Table 2:** Estimation results from negative binomial modelling on outcomes: GP visits, ED visits and hospital nights, estimates reported as incidence rate ratios.

Observations: 7,643 Clusters: 5,909	GP VISITS				ED VISITS				HOSPITAL NIGHTS			
	(1)	(2)	(3)	(4)	(1)	(2)	(3)	(4)	(1)	(2)	(3)	(4)
*E*. *coli* exceedance	1.144** **(0.062)**	1.129** **(0.059)**	1.151*** **(0.058)**	1.118** **(0.053)**	1.343** **(0.187)**	1.343** **(0.186)**	1.326** **(0.178)**	1.292* **(0.173)**	1.389** **(0.210)**	1.345** **(0.201)**	1.403** **(0.217)**	1.351** **(0.205)**
	**[1.029–1.272]**	**[1.020–1.250]**	**[1.043–1.271]**	**[1.019–1.227]**	**[1.022–1.764]**	**[1.024–1.763]**	**[1.019–1.726]**	**[0.995–1.679]**	**[1.033–1.868]**	**[1.004–1.802]**	**[1.035–1.900]**	**[1.004–1.818]**
Not mapped	1.002 (0.029)	1.020 (0.028)	1.028 (0.026)	1.031 (0.025)	0.892 (0.072)	0.924 (0.074)	0.933 (0.074)	0.935 (0.074)	1.004 (0.085)	1.026 (0.086)	1.036 (0.086)	1.039 (0.086)
Intercept	3.545*** (0.048)	1.095 (0.536)	1.161 (0.547)	2.701** (0.104)	0.171*** (0.006)	0.077* (0.110)	0.089* (0.128)	0.167 (0.241)	0.144*** (0.006)	0.010*** (0.015)	0.012*** (0.018)	0.039** (0.060)
Overdispersion parameter (ln alpha)	-0.537	-0.828	-1.015	-1.264	0.621	0.514	0.280	0.201	0.913	0.776	0.490	0.304
Log Likelihood	-18095.5	-17435.0	-17061.3	-16545.9	-3687.5	-3649.7	-3576.0	-3546.9	-3325.3	-3277.5	-3192.7	-3127.4

^a^ * p<0.1

** p<0.05

***p<0.01. (Standard errors clustered on TILDA households in parentheses). 95% Confidence intervals in square brackets.

^b^ Full model results displayed in S1 Tables 1–3 of the S1 File.

^c^ Model (1): Exceedance, not mapped.

Model (2): (1) + male, age, age squared, married, education attainment, employment status, medical card status, private health insurance status.

Model (3): (2) + self-rated health (as good or better), any difficulties with instrumental daily living, depression status (as measured by CESD-8).

Model (4): (3) + smoking status, level of physical activity, number of regular medicines category.

## 4. Discussion and conclusions

This unique study links three sources of data to examine the association between poor drinking water quality, as indicated by the detection of *E*. *coli*, on healthcare utilisation of older people. Understanding these associations can provide insights for disease prevention, healthcare management and climate adaption. A greater risk of GP visits, ED attendances and hospital nights was associated with the presence of *E*. *coli* in water supplies serving residences of older people found in this research. This analysis suggests a need to tackle water pollutants in an urgent manner to prevent a burden on healthcare. This is all the more important in the face of increasing global temperatures which present greater risks of water contamination, in combination with the ageing of developed societies.

The detection of *E*. *coli* in water supplies associated with residences occurred for a small proportion of the sample, 4%. Though, where this exceedance is modelled on the use of healthcare services, the results of this research suggest that the presence of *E*. *coli* in monitored water schemes is associated with higher demand for GP services, as well as spending nights in hospital. Therefore, we do not reject the hypothesis that the presence of waterborne *E*. *coli* leads to greater use of these healthcare services. These findings may be interpreted as concurring with studies which find that even regulated drinking water can be associated with illness, particularly for older people [4, 44], and findings which show that poor water quality is associated with greater use of healthcare, including hospital care [1, 18]. The positive result of *E*. *coli* detection on ED attendances is only marginally statistically significant, and thus the null hypothesis for this outcome cannot be rejected at the conventional significance level of p<0.05.

The linking of the three sources of information in this study–administrative water monitoring data, water systems infrastructure maps and a large, national dataset on older people’s lives–provides a novel contribution to the existing literature in this area. The availability of geo-codes for both infrastructure networks and TILDA respondents allows us to link these data together for the first time. The TILDA dataset provides rich information on the demographic, socio-economic, locational and health characteristics of older people in Ireland, which allows us to control for potential confounders in relationships between *E*. *coli* detection in water supply and healthcare utilisation.

There are a number of limitations of this research which must be acknowledged. Our data on water quality monitoring does not have *E*. *coli* results for private wells, which have the most significant problems with water contamination, and are associated with VTEC infection in Ireland [25]. In terms of the TILDA dataset, healthcare utilisation is self-reported which may be susceptible to recall bias and thus measurement error. Moreover, the survey does not ask respondents information on their water supply, behaviours around this, e.g., boiling water or use of filtration methods, nor are there greater details of illnesses e.g., acute gastrointestinal problems. The available information pertains to a single wave of TILDA and thus permits only a cross-sectional design for analysis, which does not allow confirmation of causal inferences. While we have controlled for an extensive set of possible confounding factors, it is still possible that unmeasured factors associated with both *E*. *coli* exceedance and healthcare utilisation may explain some of the findings we observe. For example, average housing quality might be relatively low in areas that also suffer from overburdened water treatment infrastructure.

This study aims to provide information relevant to policy in this area, which suggests a link between water quality and a burden on healthcare services among older people. Where drinking water is found to contribute to illness and healthcare use, this evidence can be used to support the case for intervention and action to improve the quality, treatment, and monitoring of drinking water in Ireland. The findings may be of interest to environmental and healthcare planners, the farming community, water officials, public health professionals, including the Medical Officer of Health who is legally responsible for the investigation and removal of sources of infectious diseases, in compliance with the Infectious Diseases Regulations. Considering the significant threat of climate change, there is an enhanced requirement for co-operation across all relevant agents to reduce or eliminate the health hazards presented by water contamination. Satellite surveillance data for weather and climate forecasting may become essential early warning system for water-related diseases and healthcare use [41]. Further academic study is required build a better picture in terms of the contribution of drinking water supplies, both those which are publicly regulated and private unregulated sources, to illness and the consequences and costs of this on societies in the twenty first century.

## Supporting information

S1 File The association between *E*. *coli* exceedances in drinking water supplies and healthcare utilisation of older people. (DOCX)
