# Supplementary material for: The association between E. coli exceedances in drinking water supplies and healthcare utilisation of older people
Source: PLoS One. 2022 Sep 1;17(9):e0273870. doi: 10.1371/journal.pone.0273870 (PMC9436125; doi:10.1371/journal.pone.0273870)
Supplement: S1 File — (DOCX) [file pone.0273870.s001.docx]

**Supporting Information File** **–**

The association between *E. coli* exceedances in drinking water supplies and healthcare utilisation of older people

**Map of *E. coli* detected for 2010**

**S1 Fig. 1. Map of E. coli detected across Republic of Ireland**


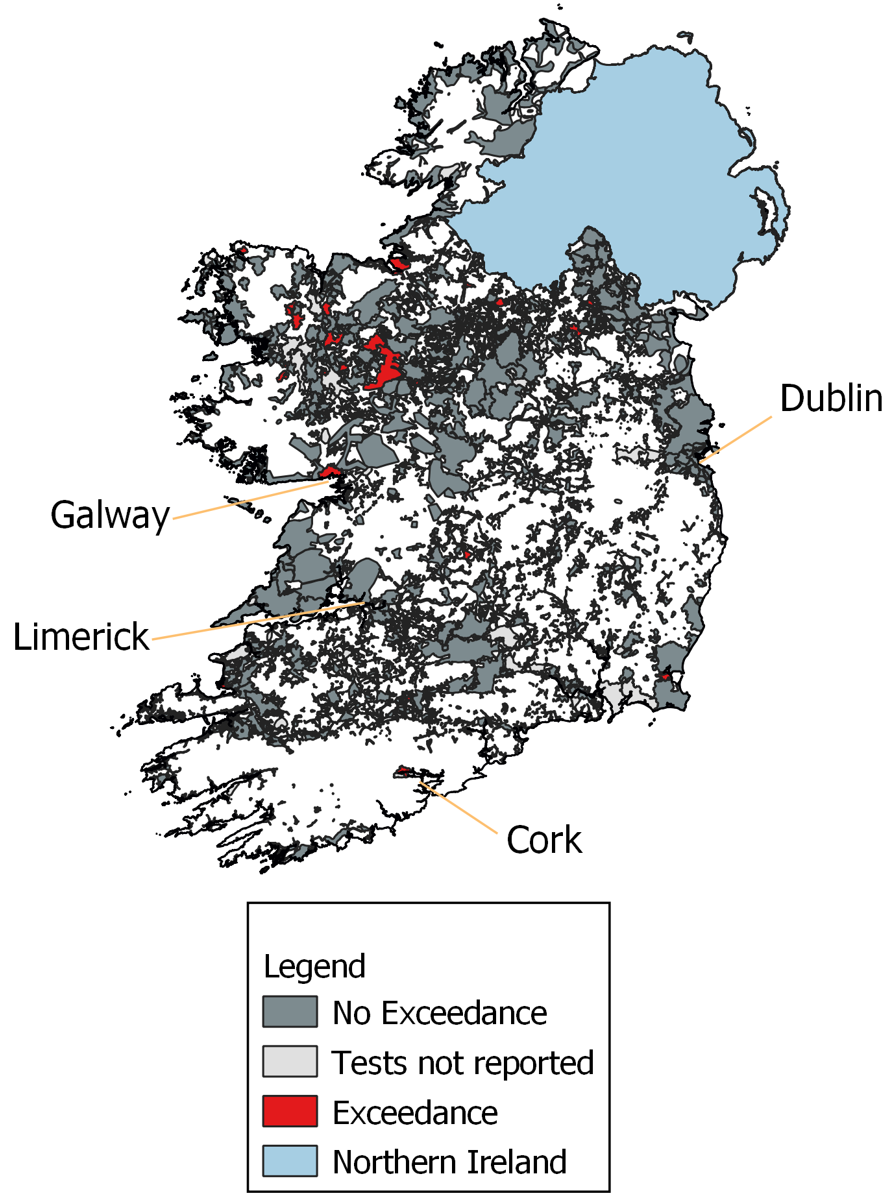


**Full modelling results**

**S1 Table 1. Estimation results from negative binomial regressions on GP visits**

| GP Visits | | | | |
| --- | --- | --- | --- | --- |
| Model | (1) | (2) | (3) | (4) |
| *E. coli* exceedance | **1.144****  **(0.062)**  **[1.029-1.272]** | **1.129****  **(0.059)**  **[1.020-1.250]** | **1.151*****  **(0.058)**  **[1.043-1.271]** | **1.118****  **(0.053)**  **[1.019-1.227]** |
| Not mapped | 1.002  (0.029) | 1.020  (0.028) | 1.028  (0.026) | 1.031  (0.025) |
| Male  *Ref: female* |  | 0.923***  (0.021) | 0.935***  (0.021) | 0.990  (0.021) |
| Age |  | 1.026*  (0.015) | 1.030**  (0.014) | 0.992  (0.013) |
| Age squared |  | 1.000  (0.000) | 1.000  (0.000) | 1.000  (0.000) |
| Married  *Ref: not married: never, married, separated, divorced, widowed* |  | 0.952**  (0.024) | 0.980  (0.023) | 0.963*  (0.022) |
| Secondary education  *Ref: primary education* |  | 0.931***  (0.025) | 0.989  (0.025) | 0.989  (0.024) |
| Tertiary education  *Ref: primary education* |  | 0.886***  (0.028) | 0.946*  (0.028) | 0.943**  (0.027) |
| Employed/self-employed  *Ref: Retired* |  | 0.866***  (0.029) | 0.931**  (0.030) | 0.984  (0.030) |
| Unemployed  *Ref: Retired* |  | 1.029  (0.064) | 1.025  (0.059) | 1.056  (0.308) |
| Looking after home/family  *Ref: Retired* |  | 0.983  (0.033) | 1.006  (0.032) | 1.016  (0.031) |
| Other employment status  *Ref: Retired* |  | 1.486***  (0.067) | 1.228***  (0.054) | 1.157***  (0.049) |
| Medical card  *Ref: No medical card* |  | 1.616***  (0.049) | 1.517***  (0.043) | 1.432***  (0.039) |
| Private health insurance  *Ref: No private health insurance* |  | 1.003  (0.026) | 1.048*  (0.026) | 1.001  (0.024) |
| Good or better self-rated health  *Ref: Fair/poor* |  |  | 0.596***  (0.015) | 0.712***  (0.018) |
| Has an instrumental activities of daily living (IADL) impairment  *Ref: No IADL impairment* |  |  | 1.211***  (0.037) | 1.123***  (0.033) |
| Depression symptoms  *Ref: No depression* |  |  | 1.236***  (0.042) | 1.189***  (0.038) |
| Smokes  *Ref: Non smoker* |  |  |  | 0.936**  (0.025) |
| Medium physical activity level  *Ref: low physical activity* |  |  |  | 0.999  (0.023) |
| High physical activity level  *Ref: low physical activity* |  |  |  | 0.921***  (0.023) |
| Number of regular medicines 1-2  *Ref: 0 medicines* |  |  |  | 1.905***  (0.062) |
| Regular medicines 3-4  *Ref: 0 medicines* |  |  |  | 2.263***  (0.079) |
| Regular medicines 4+  *Ref: 0 medicines* |  |  |  | 2.456***  (0.093) |
| Intercept | 3.545***  (0.048) | 1.095  (0.536) | 1.161  (0.547) | 2.701**  (1.243) |
| Overdispersion (ln alpha) | -0.537 | -0.828 | -1.015 | -1.264 |
| Log Likelihood | -18095.5 | -17435.0 | -17061.3 | -16545.9 |
| Observations | 7,643 | | | |
| Clusters | 5,909 | | | |

*^a^ Estimates are reported as incidence rate ratios. * p<0.1, ** p<0.05, ***p<0.01.*

*(Standard errors clustered on TILDA households in parentheses).*

*95% Confidence intervals in square brackets.*

**S1 Table 2. Estimation results from negative binomial regressions on Emergency Department visits**

| ED Visits | | | | |
| --- | --- | --- | --- | --- |
| Model | (1) | (2) | (3) | (4) |
| *E. coli* exceedance | **1.343****  **(0.187)**  **[1.022-1.764]** | **1.343****  **(0.186)**  **[1.024-1.763]** | **1.326****  **(0.178)**  **[1.019-1.726]** | **1.292***  **(0.173)**  **[0.995-1.679]** |
| Not mapped | 0.892  (0.072) | 0.924  (0.074) | 0.933  (0.074) | 0.935  (0.074) |
| Male  *Ref: female* |  | 0.943  (0.064) | 0.961  (0.064) | 1.003  (0.069) |
| Age |  | 1.030  (0.044) | 1.035  (0.044) | 1.008  (0.043) |
| Age squared |  | 1.000  (0.000) | 1.000  (0.000) | 1.000  (0.000) |
| Married  *Ref: not married: never, married, separated, divorced, widowed* |  | 0.853**  (0.060) | 0.883*  (0.062) | 0.868**  (0.061) |
| Secondary education  *Ref: primary education* |  | 0.909  (0.074) | 1.004  (0.083) | 0.996  (0.081) |
| Tertiary education  *Ref: primary education* |  | 1.078  (0.098) | 1.211**  (0.111) | 1.197**  (0.109) |
| Employed/self-employed  *Ref: Retired* |  | 0.811**  (0.081) | 0.901  (0.089) | 0.979  (0.093) |
| Unemployed  *Ref: Retired* |  | 1.038  (0.167) | 1.051  (0.168) | 1.100  (0.176) |
| Looking after home/family  *Ref: Retired* |  | 0.812*  (0.087) | 0.861  (0.092) | 0.865  (0.092) |
| Other employment status  *Ref: Retired* |  | 1.561***  (0.192) | 1.193  (0.149) | 1.131  (0.142) |
| Medical card  *Ref: No medical card* |  | 1.191**  (0.106) | 1.076  (0.095) | 1.015  (0.090) |
| Private health insurance  *Ref: No private health insurance* |  | 0.908  (0.073) | 0.972  (0.078) | 0.942  (0.076) |
| Good or better self-rated health  *Ref: Fair/poor* |  |  | 0.471***  (0.034) | 0.563***  (0.044) |
| Has an instrumental activities of daily living (IADL) impairment  *Ref: No IADL impairment* |  |  | 1.256**  (0.115) | 1.150  (0.105) |
| Depression symptoms  *Ref: No depression* |  |  | 1.297***  (0.125) | 1.251**  (0.120) |
| Smokes  *Ref: Non smoker* |  |  |  | 0.954  (0.080) |
| Medium physical activity level  *Ref: low physical activity* |  |  |  | 0.946  (0.072) |
| High physical activity level  *Ref: low physical activity* |  |  |  | 0.916  (0.076) |
| Number of regular medicines 1-2  *Ref: 0 medicines* |  |  |  | 1.511***  (0.150) |
| Regular medicines 3-4  *Ref: 0 medicines* |  |  |  | 1.783***  (0.193) |
| Regular medicines 4+  *Ref: 0 medicines* |  |  |  | 2.216***  (0.249) |
| Intercept | 0.171***  (0.006) | 0.077*  (0.110) | 0.089*  (0.128) | 0.167  (0.241) |
| Overdispersion (ln alpha) | 0.621 | 0.514 | 0.280 | 0.201 |
| Log Likelihood | -3687.5 | -3649.7 | -3576.0 | -3546.9 |
| Observations | 7,643 | | | |
| Clusters | 5,909 | | | |

*^a^ Estimates are reported as incidence rate ratios. * p<0.1, ** p<0.05, ***p<0.01.*

*(Standard errors clustered on TILDA households in parentheses).*

*95% Confidence intervals in square brackets.*

**S1 Table 3. Estimation results from negative binomial regressions on Hospital nights**

| Hospital nights | | | | |
| --- | --- | --- | --- | --- |
| Model | (1) | (2) | (3) | (4) |
| *E. coli* exceedance | **1.389****  **(0.210)**  **[1.033-1.868]** | **1.345****  **(0.201)**  **[1.004-1.802]** | **1.403****  **(0.217)**  **[1.035-1.900]** | **1.351****  **(0.205)**  **[1.004-1.818]** |
| Not mapped | 1.004  (0.085) | 1.026  (0.086) | 1.036  (0.086) | 1.039  (0.086) |
| Male  *Ref: female* |  | 1.024  (0.077) | 1.036  (0.078) | 1.123*  (0.085) |
| Age |  | 1.070  (0.047) | 1.077*  (0.047) | 1.031  (0.046) |
| Age squared |  | 1.000  (0.000) | 0.999  (0.000) | 1.000  (0.000) |
| Married  *Ref: not married: never, married, separated, divorced, widowed* |  | 0.911  (0.071) | 0.949  (0.073) | 0.921  (0.071) |
| Secondary education  *Ref: primary education* |  | 1.059  (0.090) | 1.181*  (0.100) | 1.172*  (0.098) |
| Tertiary education  *Ref: primary education* |  | 1.049  (0.104) | 1.198*  (0.118) | 1.182*  (0.112) |
| Employed/self-employed  *Ref: Retired* |  | 0.797**  (0.086) | 0.905  (0.096) | 0.970  (0.104) |
| Unemployed  *Ref: Retired* |  | 0.831  (0.151) | 0.850  (0.154) | 0.880  (0.159) |
| Looking after home/family  *Ref: Retired* |  | 0.914  (0.103) | 0.959  (0.106) | 0.951  (0.105) |
| Other employment status  *Ref: Retired* |  | 1.575***  (0.206) | 1.171  (0.155) | 1.036  (0.135) |
| Medical card  *Ref: No medical card* |  | 1.540***  (0.140) | 1.385***  (0.127) | 1.256**  (0.115) |
| Private health insurance  *Ref: No private health insurance* |  | 1.208**  (0.099) | 1.314***  (0.107) | 1.258***  (0.103) |
| Good or better self-rated health  *Ref: Fair/poor* |  |  | 0.412***  (0.032) | 0.558***  (0.046) |
| Has an instrumental activities of daily living (IADL) impairment  *Ref: No IADL impairment* |  |  | 1.370***  (0.131) | 1.172*  (0.112) |
| Depression symptoms  *Ref: No depression* |  |  | 1.166  (0.121) | 1.101  (0.112) |
| Smokes  *Ref: Non smoker* |  |  |  | 0.958  (0.092) |
| Medium physical activity level  *Ref: low physical activity* |  |  |  | 0.776***  (0.063) |
| High physical activity level  *Ref: low physical activity* |  |  |  | 0.728***  (0.068) |
| Number of regular medicines 1-2  *Ref: 0 medicines* |  |  |  | 1.688***  (0.217) |
| Regular medicines 3-4  *Ref: 0 medicines* |  |  |  | 2.538***  (0.334) |
| Regular medicines 4+  *Ref: 0 medicines* |  |  |  | 3.372***  (0.457) |
| Intercept | 0.144***  (0.006) | 0.010***  (0.015) | 0.012***  (0.018) | 0.039**  (0.060) |
| Overdispersion (ln alpha) | 0.913 | 0.776 | 0.490 | 0.304 |
| Log Likelihood | -3325.3 | -3277.5 | -3192.7 | -3127.4 |
| Observations | 7,643 | | | |
| Clusters | 5,909 | | | |

*^a^ Estimates are reported as incidence rate ratios. * p<0.1, ** p<0.05, ***p<0.01.*

*(Standard errors clustered on TILDA households in parentheses).*

*95% Confidence intervals in square brackets.*
